# *OsBBX11* on *qSTS4* links to salt tolerance at the seeding stage in *Oryza sativa* L. ssp. *Japonica*

**DOI:** 10.3389/fpls.2023.1139961

**Published:** 2023-03-08

**Authors:** Lei Lei, Liangzi Cao, Guohua Ding, Jinsong Zhou, Yu Luo, Liangming Bai, Tianshu Xia, Lei Chen, Jiangxu Wang, Kai Liu, Qingjun Lei, Tingting Xie, Guang Yang, Xueyang Wang, Shichen Sun, Yongcai Lai

**Affiliations:** ^1^ Postdoctoral Scientific Research Station of Heilongjiang Academy of Agricultural Sciences, Harbin, China; ^2^ Institute of Crop Cultivation and Tillage, Heilongjiang Academy of Agricultural Sciences, Harbin, China; ^3^ Heilongjiang Rice Quality Improvement and Genetic Breeding Engineering Research Center, Harbin, China; ^4^ Northeast of National Center of Technology Innovation for Saline-Alkali Tolerant Rice, Harbin, China; ^5^ Heilongjiang Academy of Agricultural Sciences, Harbin, China; ^6^ Branch of Animal Husbandry and Veterinary of Heilongjiang Academy of Agricultural Sciences, Qiqihar, China

**Keywords:** japonica rice, seedling stage, salt tolerance, b-box zinc finger family protein, function characterization

## Abstract

Rice has been reported to be highly sensitive to salt stress at the seedling stage. However, the lack of target genes that can be used for improving salt tolerance has resulted in several saline soils unsuitable for cultivation and planting. To characterize new salt-tolerant genes, we used 1,002 F_2:3_ populations derived from Teng-Xi144 and Long-Dao19 crosses as the phenotypic source to systematically characterize seedlings’ survival days and ion concentration under salt stress. Utilizing QTL-seq resequencing technology and a high-density linkage map based on 4,326 SNP markers, we identified qSTS4 as a major QTL influencing seedling salt tolerance, which accounted for 33.14% of the phenotypic variation. Through functional annotation, variation detection and qRT-PCR analysis of genes within 46.9 Kb of *qSTS4*, it was revealed that there was one SNP in the promoter region of *OsBBX11*, which resulted in a significant response difference between the two parents to salt stress. Transgenic plants using knockout-based technology and demonstrated that Na^+^ and K^+^ in the roots of the functional-loss-type *OsBBX11* were translocated largely to the leaves under 120 mmol/L NaCl compared with the wild-type, causing *osbbx11* leaves to die after 12 days of salt stress due to an imbalance in osmotic pressure. In conclusion, this study identified *OsBBX11* as a salt-tolerance gene, and one SNPs in the *OsBBX11* promoter region can be used to identify its interacting transcription factors. This provides a theoretical basis for finding the molecular mechanism of *OsBBX11* upstream and downstream regulation of salt tolerance and molecular design breeding in the future.

## Introduction

Rice (Oryza sativa L.) is a staple food for much of the global population (Ponce et al., 2021), Approximately 30% of the global rice cultivation area is affected by salinity (Takehisa et al., 2004). According to the data from the United Nations Educational, Scientific and Cultural Organization and the United Nations Food and Agriculture Organization, China has 9913 hectares of saline-alkali land (He and Luo, 2021). When the concentration of soluble salt in the soil reaches 0.3%, the photosynthetic efficiency of rice seedlings decreases, and the organism exhibits ionic imbalance and high osmotic pressure, leading to plant wilting and apoptosis, and reduced rice yield (Akbar et al., 1972; Ma et al., 2017), which severely restricts the distribution of rice planting. Therefore, screening salt-tolerant rice varieties and analyzing the genetic mechanism of salt tolerance in rice are of great significance for ensuring rice production.

Salt tolerance of rice is a typical quantitative trait controlled by multiple genes (Johnson et al., 1992). To date, approximately one thousand QTLs associated with salt tolerance in different growth stages of rice have been reported (Prakash et al., 2022). However, most of the QTLs are difficult to utilize in molecular marker-assisted breeding, leading to only *qSKC-1* and Saltol (Lin et al., 2004; Ren et al., 2005) being finely mapped or map-cloned, and the candidate gene for Saltol has yet to be isolated. Therefore, it is highly challenging to clone salt-tolerance QTLs in rice. Reverse genetic approaches have confirmed that more than 300 rice genes, including transcription factors *OsbZIP20* (Baoxiang et al., 2022), *OsbZIP72* (Baoxiang et al., 2021), *OsMYB106* (Wang et al., 2020), ion transporter protein *OsHKT2;2* (Kader et al., 2006), *OsNHX2* (Fukuda et al., 2011), protein kinase *SIT1*/*SIT2* (Li et al., 2014), and *OsMAPKKK63* (Na et al., 2019), regulate salt tolerance in rice. However, transgenic salt-tolerant rice typically displays growth deficiencies and lacks genetic salt-tolerant allele data, making it difficult to utilize for rice molecular breeding. Molecular marker-assisted selection breeding can reduce the duration of salt-tolerant rice breeding by four to seven years, with Saltol being the most widely employed. (Bimpong et al., 2016), only *Saltol* has been extensively used in salt tolerance breeding (Salam et al., 2007; Singh et al., 2011; Babu et al., 2012; Linh et al., 2012; Gregorio et al., 2013; Vinod et al., 2013). However, its utilization is restricted to India, the Philippines, Bangladesh, Thailand, Vietnam, and Senegal(Linh et al., 2012; Singh et al., 2016), and no salt-tolerant genes suitable for application in the Chinese rice cultivation context have been identified. Consequently, it is imperative to conduct further research and uncover salt-tolerant QTL/genes for potential functional applications in China.

In comparison to traditional QTL mapping, the integration of QTL-Seq technology and high-density linkage maps can expedite the identification of major genes of rice traits(Kodama et al., 2018; Sun et al., 2019). Additionally, resistance genes can be extracted from local cultivated varieties and utilized for molecular breeding, taking advantage of its superior allelic variations without the need to consider the genetic background interference caused by kinship (Xiao et al., 2018; Xu et al., 2020), thus yielding salt-tolerant varieties suitable for production. Nevertheless, the salt-tolerant QTLs reported mainly originate from the varieties developed by the International Rice Research Institute and the indica or japonica rice in southern China, while few are from northern China. In the present study, we used a QTL-Seq and high-density linkage mapping approach with 1002 F_2_ populations derived from a cross between Teng-Xi144 (TX144, a salt-sensitive variety) and Long-Dao19 (LD19, a strongly salt-tolerant variety) and five traits: survival days of seedlings (SDSs), the shoot Na+ concentration (SNC), shoot K+ concentration (SKC), root Na+ concentration (RNC), and root K+ concentration (RKC) under 120mmol/L NaCl stress, to identify major QTL intervals for salt tolerance at the seedling stage (STS) in rice. The candidate genes of QTL were identified by detecting the variation of sequence and the expression level of genes under salt stress. Next, the salt tolerance function of the gene was characterized by the salt tolerance phenotype of the transgenic plants. This provides a key theory for the molecular breeding of salt tolerance genes and the molecular mechanism of salt tolerance in Longdao 19.

## Materials and methods

### Plant materials

For QTL-sequencing and linkage mapping, the two extreme rice cultivars, namely, TX144 (salt-sensitive) as the female parent and LD19 (salt-tolerant) as the male parent, were crossed to develop a population of 1002 F_2:3_ by single seed descent at the experimental station of Heilongjiang Academy of Agricultural Sciences (Heilongjiang Province, China; 47°98′ N, 128°08′ E; 128 m above sea level).

### Evaluation of salt tolerance at the seedling stage

Salt tolerance for all 1002 F_2:3_ and their parents was evaluated in a hydroponics system at the experimental station of Northeast Agricultural University. Briefly, the individuals were harvested and kept at 40°C for 2 days to break seed dormancy. Afterward, the seeds were surface-sterilized with 2% NaClO_3_ for 10 min, washed thrice with sterile water, and soaked in water in Petri dishes at 30°C for germination. When the seed bud was equal to half the length of the seed, 50 uniformly germinated seeds were selected in each Petri dish and hydroponically grown in the Yoshida nutrient solution. Each experimental treatment was repeated thrice. The climatic cabinet temperature was set at 28°C/25°C day/night cycle with 60% relative humidity. Rice reached the three-leaf stage and was treated with 120 mmol/L NaCl solution in a stressful environment for 7 days, following which the salt solution was replaced with nutrient solution, and the incubation was continued for 5 days. Next, survival days of seedlings (SDSs) were recorded for each plant in days from seeding to death. To observe physiological traits, the second experiment was performed. The procedure and administration of the experiments were the same as those described above. At 7 days after treatment with NaCl, each sample’s shoots were harvested and dried at 100°C for 30 min and 60°C for 1 week. A total of 0.1 g of dried sample was ground and subsequently digested with 0.1 N nitric acid (Fisher Scientific) at 70°C for 8 h (Campbell et al., 2017). The shoot Na^+^ concentration (SNC), shoot K^+^ concentration (SKC), root Na^+^ concentration (RNC), and root K^+^ concentration (RKC) were measured using a flame photometer (Sherwood 410, Cambridge, UK) to evaluate the salt tolerance at the seedling stage.

### QTL-seq for salt tolerance

To prepare paired-end libraries with an insert size of 500 bp using the paired-end DNA sample prep kit (Illumina, San Diego, CA, USA), the total genomic DNA was extracted from bulked pools, and at least 3 g of the genomic DNA were used to construct genomic libraries. A HiSeq X10 (Illumina) NGS platform was used at Gene Denovo (Guangzhou, China) to sequence these libraries. Quality trimming is an essential step in generating high-confidence variant calling. To achieve high-quality clean reads, we used the following three stringent criteria to filter the raw reads: 1) removing reads with more than 10% unidentified nucleotides; 2) removing reads with > 50% PhRED quality scores of > 20; and 3) aligning reads to barcodes.

Burrows–Wheeler Aligner (v.0.7.16a-r1181) was used to align filtered reads with the Nipponbare reference genome sequence (Matsumoto et al., 2005) using the parameter ‘mem-M’. Using mem-M, shorter split-alignment hits were marked as secondary alignments (Kumar et al., 2019). UnifiedGenotyper (v.3.5) was used for variant calling (http://gatk.broadinstitute.org/hc/en-us/community/posts/36007363711-0-UnifiedGenotyper-in-GATK4). In the ANNOVAR tool (Wang et al., 2010), all mutations were annotated for genes, functions, and genomic regions. SNP-Index (Takagi et al., 2013), G-Statistic (Magwene et al., 2011; Mansfeld and Grumet, 2018), ED (Hill et al., 2013) and two-tailed Fisher’s exact test (Fisher, 1922) based on SNPs were used for association analysis and calculated using a 2000-kb sliding window with a step size of 20 kb and excluding windows with less than 10 SNP/Indel. The results obtained were merged into the next window in case of insufficient SNPs. The final QTL interval was calculated using the overlapping intervals of the four methods. The raw Illumina sequencing data from this study were submitted to the NCBI’s Sequence Read Archive (SRA) database under accession number SRR22671405, SRR22671406, SRR22671407, and SRR22671408.

### Construction of genetic linkage mapping and QTL mapping for salt tolerance

To extract the genomic DNA, Fresh leaflets 3 to 5 cm in length were collected and ground, and frozen in liquid nitrogen (Promega Corporation, Madison, WI, USA). SNP assays were conducted using DNA samples showing high quality (minimum 10 kb fragments) and an appropriate concentration (10–50 ng/mL) according to agarose gel electrophoresis. Only high-quality DNA samples were used for SNP analysis. Polymorphism in 1002 F_2:3_ lines and their parents was detected using a 10 K rice genotyping by target sequencing (GBTS) liquid phase chip based on GenioBits technology (Molbreeding Biotechnology Co., Shijiazhuang, China). We first screened SNPs according to parental genotypes, and afterward removed redundant markers from datasets using the IciMapping 4.2 software’s “BIN” computing module. Finally, these BINs were mapped using the JoinMap 4.0 software (Van Ooijen, 2006). QTL analysis was based on the arithmetic mean values of three replicates for each trait, determined by the inclusive composite interval mapping in IciMapping 4.2. LOD ≥ 2.5 was set as the threshold to decide the QTL for each trait, with 1 cM walking speed.

### RNA extraction and qRT-PCR analysis

At the two-leaf stage, plants in the control experiment were grown under normal conditions, and seedlings in the stress experiment were exposed to salt stress (120 mM NaCl). Leaf samples were collected and rice leaves from TX144 and LD19 were sampled from both sets of experiments at 0 and 12 h of stress and immediately frozen in liquid nitrogen. Next, 2 g of the total RNA was extracted using the TRIzol reagent, and first-strand cDNA was synthesized using Invitrogen’s Superscript II Reverse Transcriptase kit (Invitrogen). The qRT-PCR was performed using a Roche Light Cycler 2.10 system and the 2Fast qPCR Master Mix. Primer 5.0 software was used to develop gene-specific primer sequences (**Supplementary Table 1**). Three biological replicates and three technical replicates were used for measurements. *Actin-EFα1* (LOC_Os03g08010) was used as the internal control (Livak and Schmittgen, 2001). The relative gene expression was calculated using the 2^-ΔΔCt^ method (Livak and Schmittgen, 2001).

### Generation of transgenic rice plants

To generate *qsts4* knockdown plants, plasmids were introduced into the *Agrobacterium tumefaciens* strain EHA105. Plasmid construction and plant transformation were performed as described previously (Li et al., 2017). Two target sequences, including point accepted mutation (PAM) (GGTGTGCTGCGCGGACGAGGCGG/GGCGTTCATCTTCTGCGTGGAGG) were selected within the target genes. After PCR-mediated DNA amplification in the knockout lines, the designed target amplicons (300-500 bp) were sequenced directly and identified using degenerate sequence decoding (Ma et al., 2015). Knockout lines were detected by PCR sequencing with primers 5’-TGTGCGGTTTTGAGGGGAGT-3’ and 5’-TAATCTTGTTATCAAGAATG-3’. Primers used for vector construction are listed in **Supplementary Table 1**.

### Statistical analysis

The descriptive statistical analysis and differences between parental and F_2:3_ lines for salt tolerance were performed using the SigmaPlot software (v.12.5; Systat Software, San Jose, CA, USA). Data represent the mean ± standard deviation. All graphs were drawn using Origin 2018 (OriginLab, Northampton, MA, USA) or SPSS 18.0 (IBM, Armonk, NY, USA).

## Results

### Screening and evaluation of salt tolerance at the seedling stage

**Figure 1:**
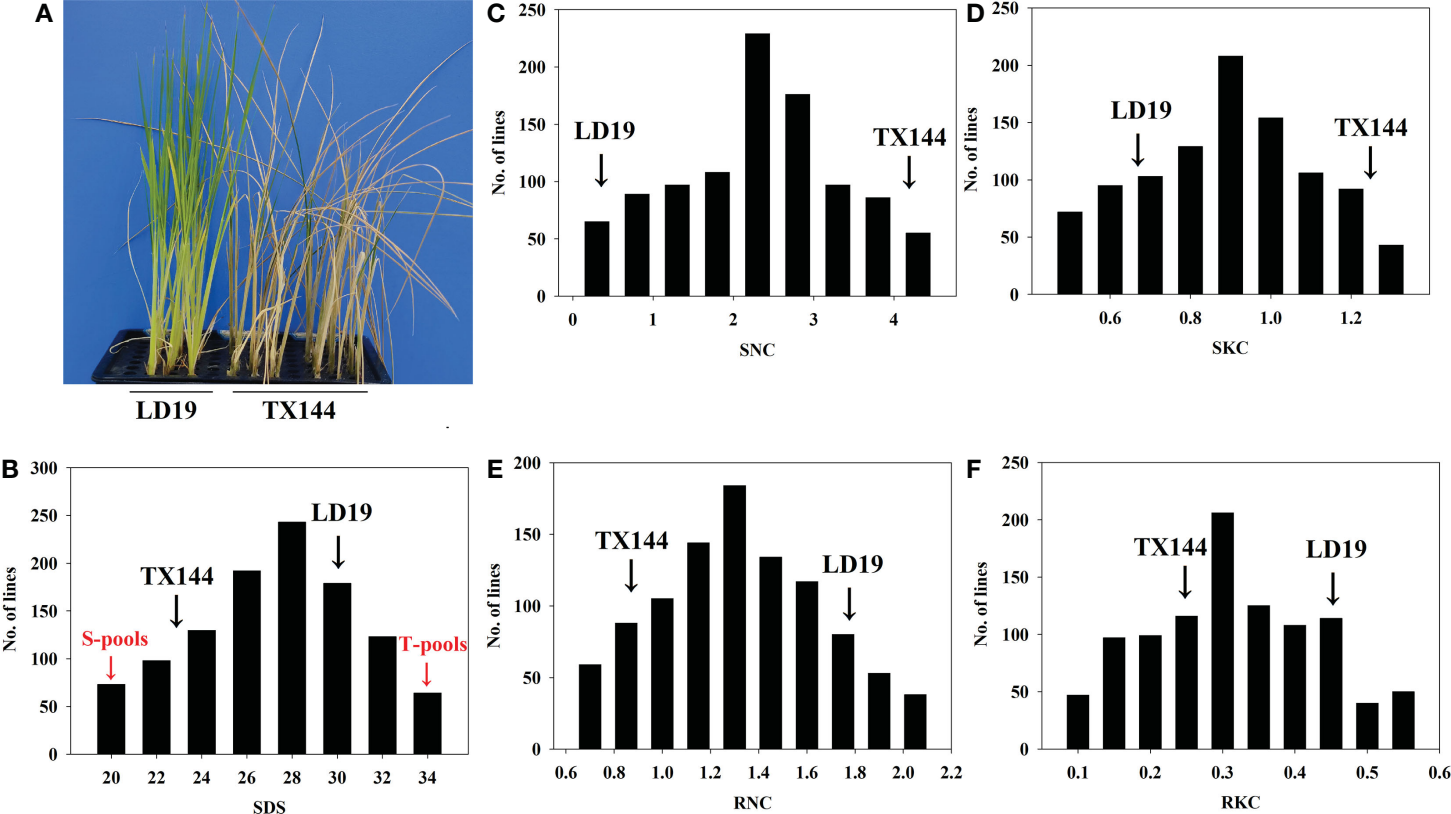
Phenotypic analysis of the parents and their derived populations under salt stress. **(A)** Comparison of the growth of seedling plants of the two parents under salt stress. **(B–F)** Frequency distribution of days of seedling survival and four physiological traits of shoots and roots of 1002 F_2:3_ lines under salt stress.

We assessed the phenotypic variations under salt stress and control conditions. Out of two parental varieties, TX144 but not LD19, presented a salt-sensitive phenotype, indicating that LD19 had stronger salt tolerance (**Figure 1A**). In addition, survival days of seedlings (SDS) of 1002 F2:3 progeny (**Figure 1B**) ranged from 20 to 34 days, of which 50 most and least salt-tolerant progeny were assigned to T-pools and S-pools, respectively, for DNA re-sequencing. To study physiological traits under salt stress, four traits were measured. The F_2:3_ population showed continuous segregation. The SNC, SKC, RKC, and RNC demonstrated a normal distribution (**Figures 1C–F**), which was consistent with the inheritance of quantitative traits. Moreover, the absolute values of skewness and kurtosis for all traits were < 1, indicating that the data of these traits were suitable for QTL analysis (**Supplementary Table 2**).

### Correlation among physiological traits

Pearson correlation coefficients were used to assess the correlation between different traits. **Supplementary Table 3** shows a significant correlation among the five traits under salt stress. SDS was only negatively correlated with SNC in the shoots, whereas SNC was negatively correlated with SKC in the shoots. We observed a positive correlation between RNC and between RKC for both traits in seedling roots, suggesting a more significant influence of these traits under salt conditions.

### QTL-seq analysis

**Figure 2:**
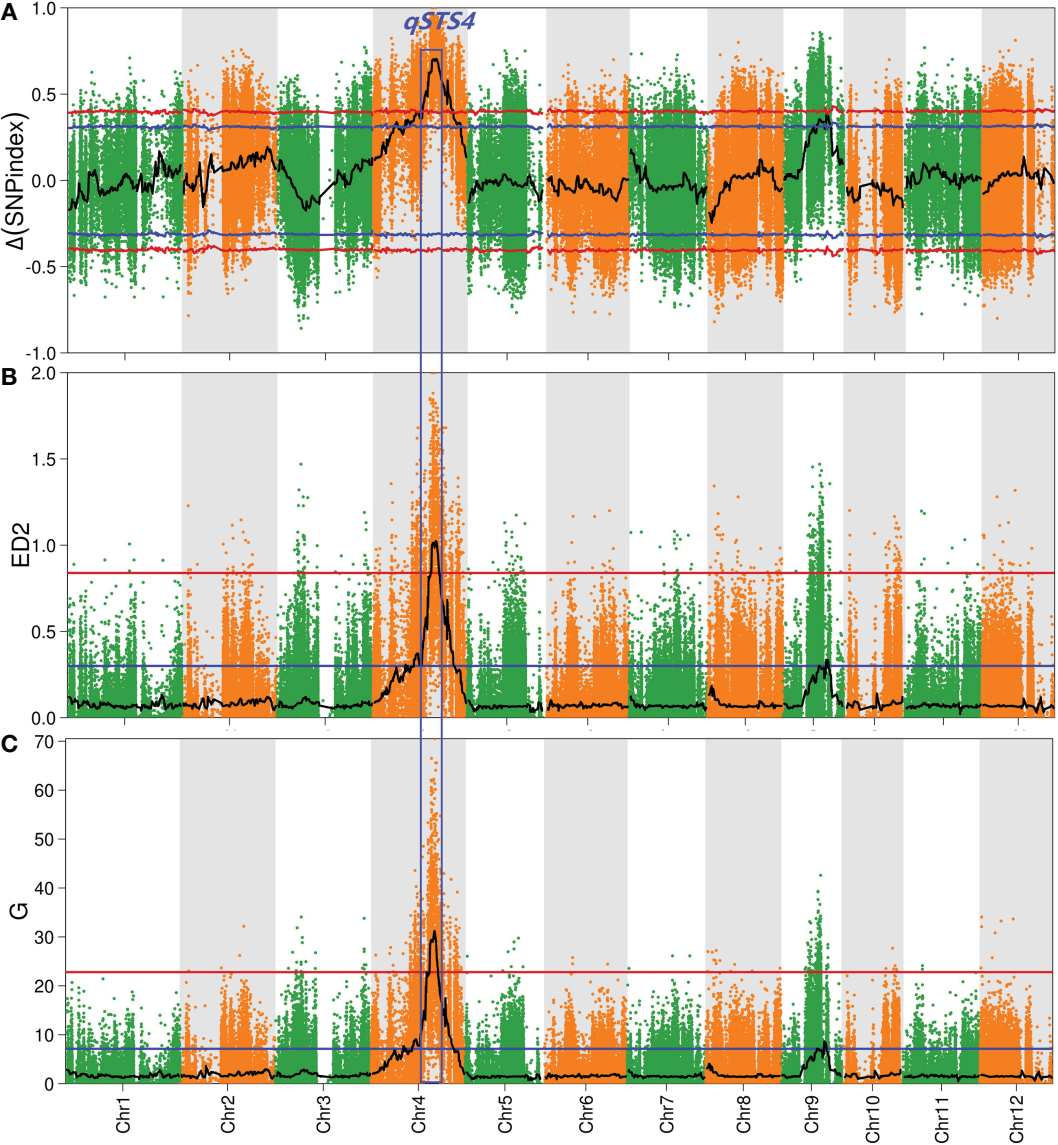
Quantitative trait locus (QTL) analysis of rice salt tolerance at seeding stage by QTL-seq. **(A–C)** represent Manhattan plots of Δ (SNP-index), Euclidean distance (ED2) and G-value on chromosomes, respectively. The blue and red lines represent the 95% and 99% confidence intervals, respectively, and the black line represents the mean of the four algorithms, plotted using sliding window analysis. The numbers on the horizontal coordinates represent chromosome numbers.

The average sequencing depth of parents and the two pools was 50×. In addition, 1,067,436 SNPs and 177,492 indels were blasted using the ‘Nipponbare’ reference genome, followed by trimming and filtering to finally generate 443,872 SNPs and 72,173 indels. Finally, 516,045 high-quality SNPs/indels were preferentially selected for QTL-sequencing analysis (**Supplementary Table 4**). Δ (SNP-Index) values (Takagi et al., 2013), Euclidean distance (ED) values (Hill et al., 2013), G values (Magwene et al., 2011; Mansfeld and Grumet, 2018), and Fisher’s exact test *P*-values (Fisher, 1922) were used to identify the candidate QTL regions contributing to salt tolerance at the seedling stage (STS). **Figure 2** and **Supplementary Table 5** show that only a significant (*P* < 0.01) peak in the Δ (SNP-Index), ED, and G values, whereas E distributions spanned the intervals of 14.4-Mb (16.3-30.7 Mb), 4.2-Mb (21.2-25.4 Mb), and 5.2 (20.4-25.6 Mb) on chromosome 4, respectively. We designated this QTL as *qSDS4*. Altogether, a 4.2-Mb overlapping interval of the three methods resulted in the final *qSTS4* interval.

### Linkage mapping analysis

**Figure 3:**
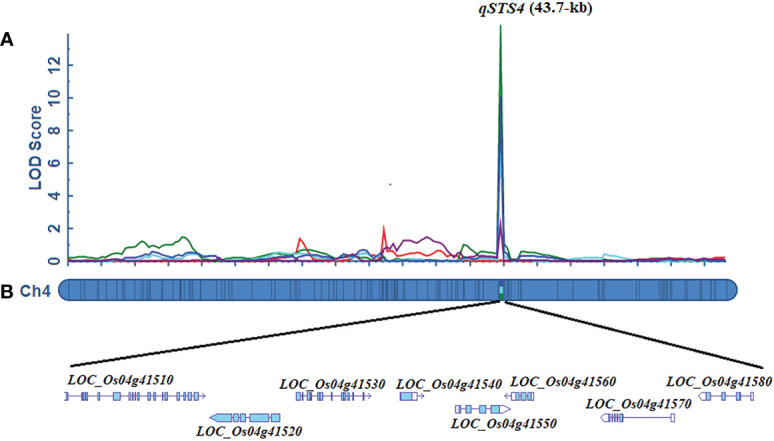
Fine mapping of the qSTS4. **(A)** Localization of qSTS4 based on high-density mapping by ICIM module of QTL IciMapping 4.2. **(B)** Putative salt genes identified at qSTS4 using annotation information from the ‘Nipponbare’ reference genome (http://plants.ensembl.org/index.html/).

The 10K rice genotyping chip was used to detect 4,326 SNPs in 1002 F_2:3_ lines and their parents. A total of 1,107 bin markers were evenly distributed on 12 chromosomes and obtained using the “BIN” calculation module of the IciMapping 4.2 software. A linkage map covering a total genetic distance of 2252.63 cM of the rice genome was obtained, and the average distance between Bin markers was 2.03 cM (**Figure S1**). For SDS, two QTLs were detected on chromosomes 1 and 4, respectively (**Supplementary Table 6**; **Figure S1**). *qSDS4-1* which exerted the most significant effect contributed to 13.89% of the total phenotypic variance. The additive effect of the LD19 allele increased SDS by 3.20 days. The other QTLs, such as qSDS1, accounted for 6.0% of the total phenotypic variance, and the LD19 allele at *qSDS1* increased the SDS (**Supplementary Table 6**). For SKC, SNC, and RKC, a significant LOD peak interval on chromosome 4 was detected. We designated these QTLs as *qSKC4, qSNC4, and qRKC4*, respectively. The percentages of phenotypic variance explained by *qSKC4, qSNC4, and qRKC4* were 29.21%, 30.40%, and 28.09%, respectively. For RNC, only qRNC was detected on chromosome 4. This QTL had a relatively small effect, which contributed to 8.12% of the total phenotypic variance (**Supplementary Table 6**). Altogether, *qSDS4-1*, *qSKC4, qSNC4*, and *qRKC4* were anchored within the 46.9-Kb interval between 24625017-bp and 24671947-bp, which overlapped completely with the *qSDS4-1* interval detected by QTL-sequencing. Therefore, this peak interval with one-causal multiple effects was considered the STS QTL and termed *qSTS4* (**Figure 3A**).

### Identification of candidate genes for qSTS4

**Figure 4:**
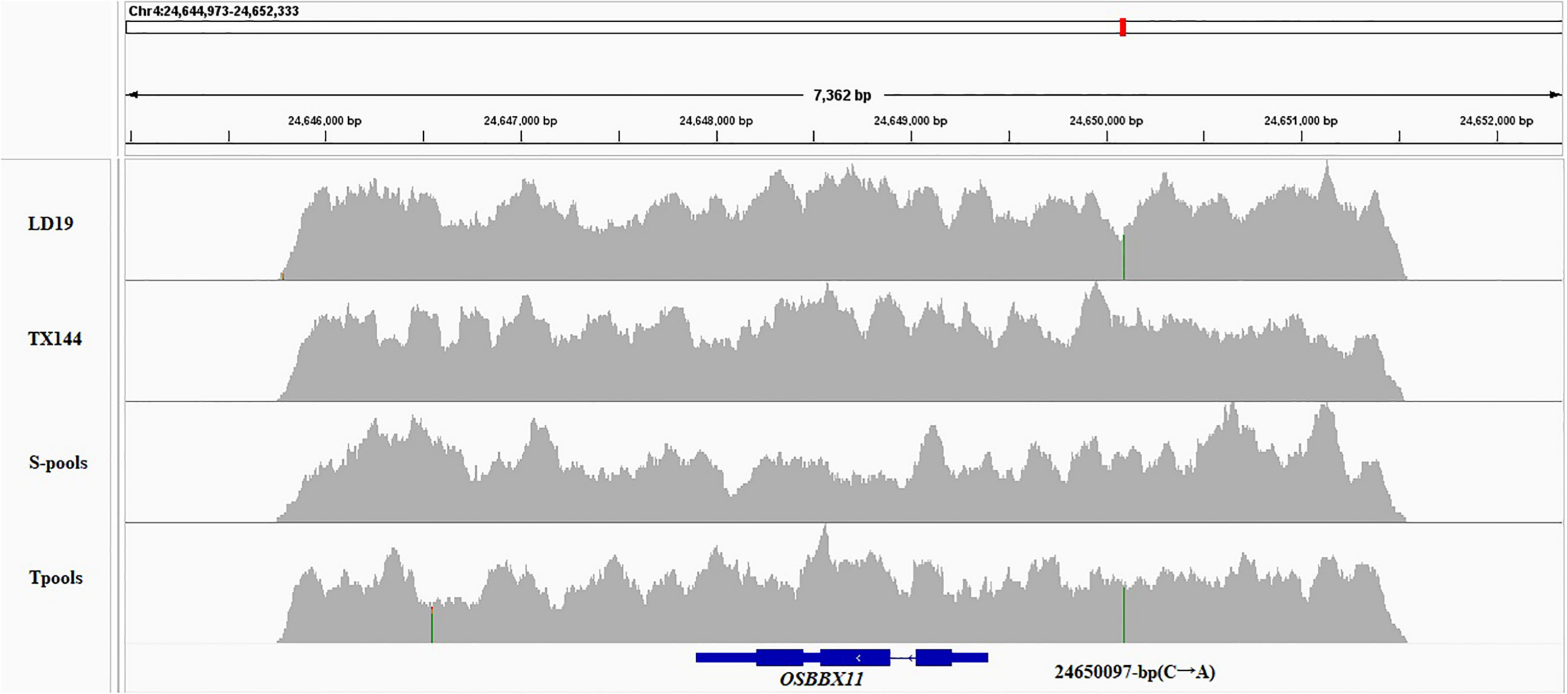
Visualization of variation information of OsBBX11 in four samples based on IGV.

**Figure 5:**
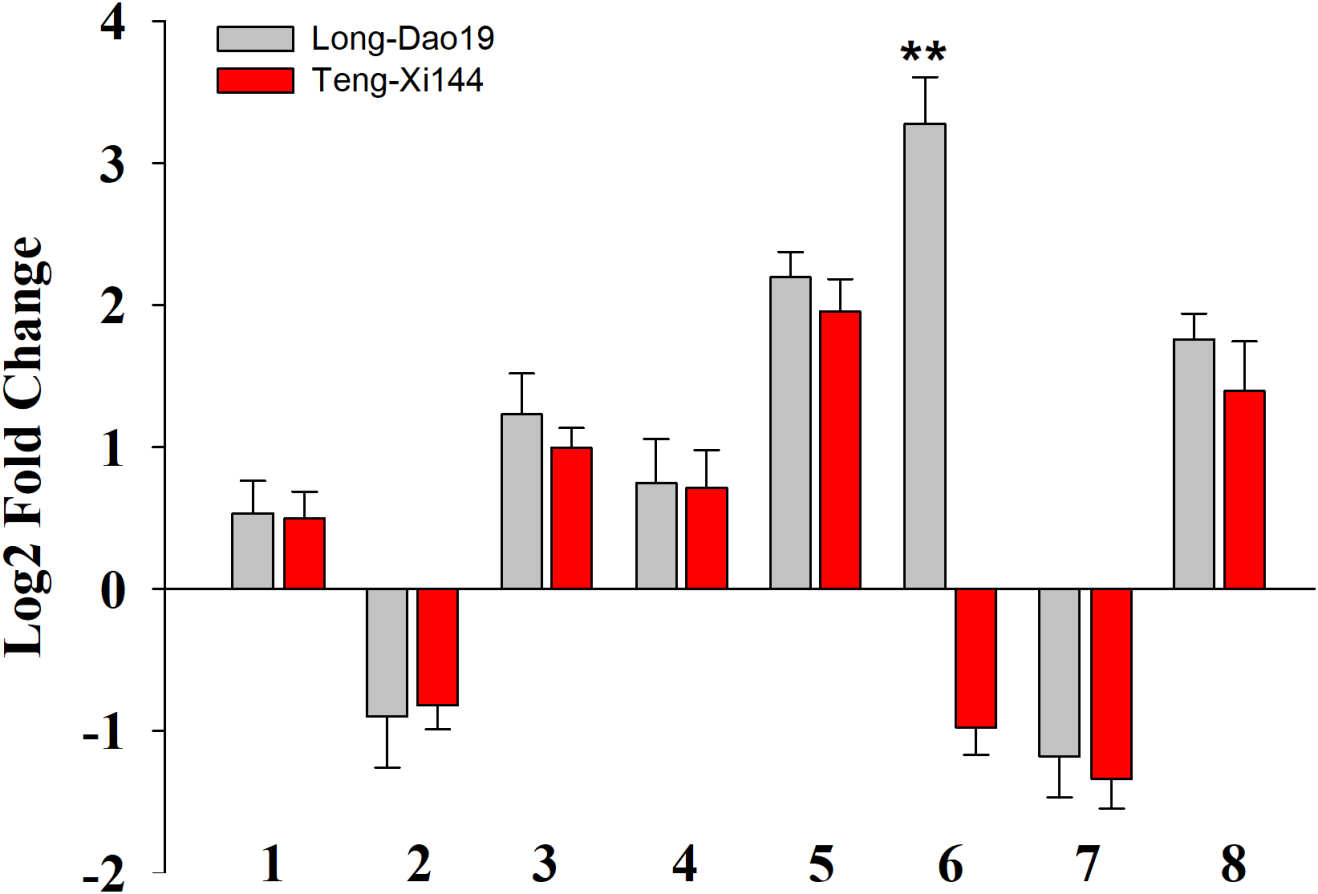
The expression level of selected genes in LD19 and TX144 12h after exposure to salt stress. Log2-fold change was calculated for gene expression under salt stress compared with control. 1–8 represents the genes used for expression analysis. 1-LOC_Os04g41510; 2-LOC_Os04g41520; 3-LOC_Os04g41530; 4-LOC_Os04g41540; 5-LOC_Os04g41550; 6-LOC_Os04g41560; 7-LOC_Os04g41570; 8-LOC_Os04g41580. **P < 0.01; Student’s t-test.

Based on the Nipponbare genome annotation (http://plants.ensembl.org/index.html/), the annotation information of the eight genes within the *qSTS4* interval is shown in **Supplemental Table 7** and **Figure 3B**. These genes include 1 retrotransposon protein, 2 serine/threonine-protein kinase GCN2, 1 CRS1/YhbY domain-containing protein, 1 B-box zinc finger family protein, 1 ethylene-responsive protein-related, 1 glycine-rich protein, and 1 Calmodulin-related calcium sensor protein. Out of the eight annotated genes, only LOC_Os04g41560 was a B-box zinc finger family protein (*OsBXX11*), which was annotated as the function of “response to abiotic stimulus (GO:0009628, GO:0009791).” Orthologous group of LOC_Os04g41560 in plants, AT2G31380 in *Arabidopsis*, Sb04g025400 and Sb06g021170 in *Sorghum*, GRMZM2G018876, GRMZM2G019335, and GRMZM2G095299 in maize, and Bradi3g48180 in *Brachypodium* were annotated as putative salt tolerance genes using the http://rice.uga.edu/ database. Moreover, two of the eight annotated genes contained two SNP loci in the upstream or CDS, including one SNP in the upstream LOC_Os04g41560 (**Figure 4**) and one SNP in the CDS of LOC_Os04g41520 (**Supplemental Table 8**). To verify the expression characteristics of eight genes under salt stress, their expression patterns under salt stress and control (no stress) were determined by real-time reverse transcription PCR (qRT-PCR). Under salt stress, LOC_Os04g41520 and LOC_Os04g41570 were downregulated in both LD19 and TX144, and LOC_Os04g41510, LOC_Os04g41530, LOC_Os04g41540, LOC_Os04g41550, and LOC_Os04g41580 were up-regulated in both LD19 and TX144. Compared to TX144, only LOC_Os04g41560 was upregulated in LD19 under salt stress (**Figure 5**), implicating LOC_Os04g41560 in regulating salt tolerance in rice.

### Characterization of salt tolerance of LOC_Os04g41560 using knockout plants

**Figure 6:**
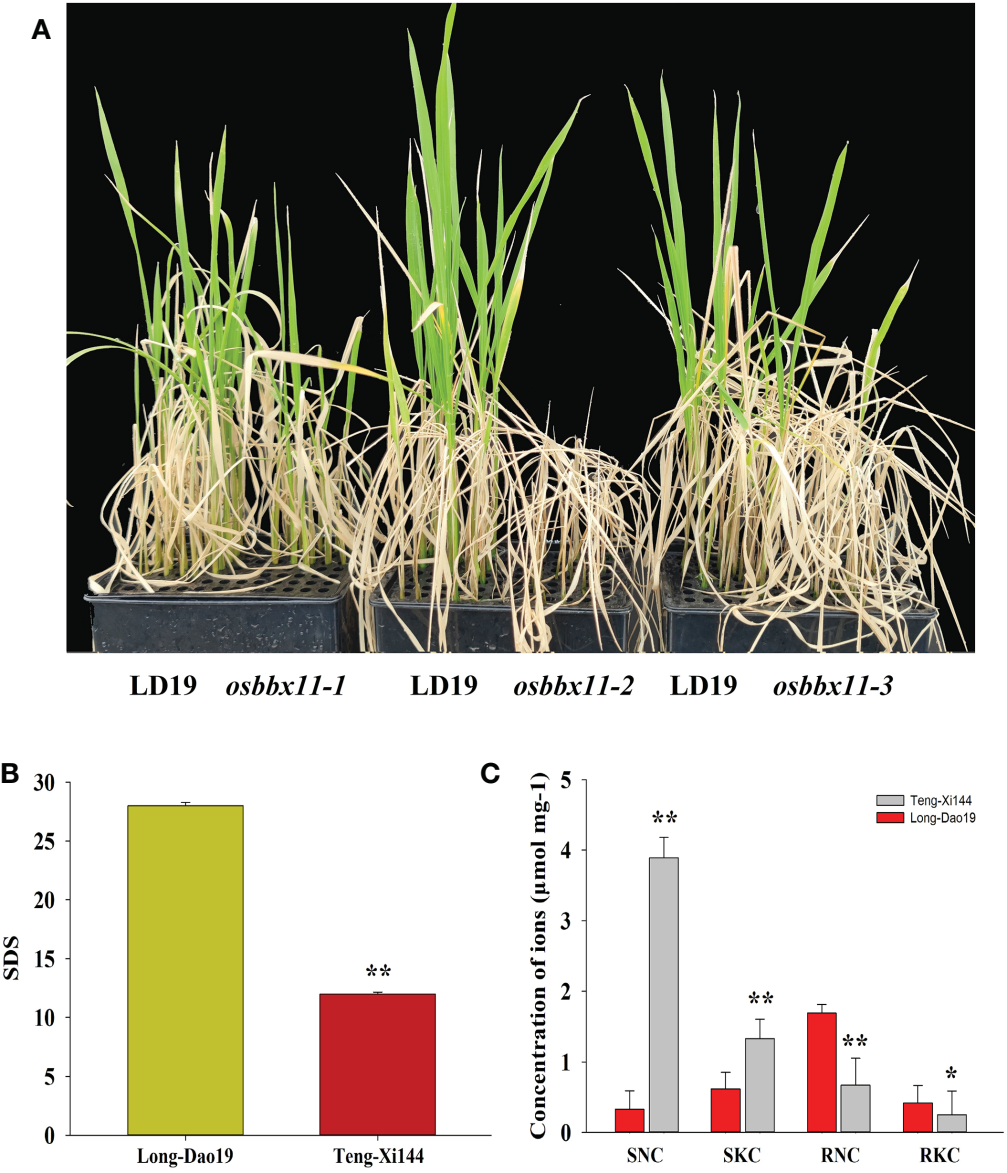
Functional analysis of rice’s B-box zinc finger family protein LOC_Os04g41560 (*OsBX11*). **(A)** Salt tolerance phenotype of knockout plants after 10 days of salt stress and 12 days of resuming growth in a non-salt environment. **(B)** Comparison of seedling survival days after salt stress between knockout and wild-type plants. **(C)** Comparison of Na^+1^ and K^+1^ concentrations in roots and shoots of knockout and wild-type plants after salt stress. **P < 0.01; Student’s t-test *P < 0.05; Student’s t-test.

Next, we verified the salt tolerance of LOC_Os04g41560 (*OsBXX11*) using knockout lines generated by clustered regularly interspaced short palindromic repeats (CRISPR)/CRISPR-associated nuclease 9 (Cas9)-mediated gene editing. As shown in **Figure 6A**, *osbbx11* exhibited a strong loss-of-function phenotype under salt stress, i.e., almost all seedlings died after 10 days of salt stress and 12 days of resuming growth in a non-salt environment (**Figure 6B**). Subsequently, we collected these rice seedlings and measured the SNC, SKC, RNC, and RKC. The results showed that the SNC and SKC in *osbbx11* shoots were more than two times higher than in LD19 shoots. In contrast, the values of RNC and RKC in the roots of *osbbx11* were low compared with those of LD19 (**Figure 6C**). Altogether, *OsBBX11* has a positive and significant function in STS.

## Discussion

Salt tolerance at the seedling stage is highly crucial because the effects on initial plant stages decide the production (Liang et al., 2015). Therefore, it is essential to decipher the mechanism of salt tolerance at the seedling stage to improve rice yield. Although several QTLs and candidate genes contributing to seedling salt tolerance have been identified in rice(Nayyeripasand et al., 2021; Yadav et al., 2021; de Ocampo et al., 2022; Yuan et al., 2022). To date, the molecular mechanism of salt tolerance in rice at the seedling stage has not been elucidated. Several factors, such as chlorophyll contents, Na^+^ uptake, K^+^ uptake, ionic balance, and ionic compartment contribute to salt tolerance (Nieves-Cordones et al., 2016; Le et al., 2021; Nakhla et al., 2021; Tian et al., 2021).STS was regulated by the balanced relationship between Na^+^ and K^+^ in the roots and shoots. As shown in **Figure 1**, in response to salt stress, the salt-sensitive variety TX144 excessively accumulated Na^+^ and K^+^ in the leaves compared with the salt-tolerant variety LD19. However, the ion concentration in the roots of TX144 was reduced, and it could survive for only 23 days. Furthermore, Several studies have demonstrated that the uptake of Na^+^ and K^+^ by rice is independent under salt stress (Yeo et al., 1987; Koyama et al., 2001). However, the data in **Supplemental Table 3** reveals a negative correlation between SKC and SNC, indicating competition between Na^+^ and K^+^ in the shoots. However, in the roots, the relationship between Na^+^ and K^+^ was synergistic. For related traits, QTLs were often mapped in the same chromosomal region (Paterson et al., 1991; Lin et al., 2004; Pundir et al., 2021). We found that *qSTS4* was linked to SDS, SNC, SKC, and RKC. These results suggest that the correlation of traits could be attributed to polymorphic effects or close linkage of genes. In addition, the results indicate a vital function of *qSTS4* in determining salt tolerance in both parental genotypes.

It is highly challenging to develop high-density genetic maps and a series of near-isogenic lines using traditional map-based QTL cloning methods. QTL-sequencing has merged as a powerful tool to rapidly detect major QTLs. Moreover, it has been applied to the genetic study of salt tolerance in crops; however, these studies reveal hundreds of candidate genes in the QTL interval that need to be researched (Tiwari et al., 2016; Shamaya et al., 2017; Kodama et al., 2018; Wu et al., 2020). An alternative method to identify candidate genes is to design molecular markers within the interval obtained by QTL-sequencing and further narrow QTL intervals through linkage maps. (Zhang et al., 2018). However, because salt tolerance is a complex quantitative inheritance in crops, the difference interval of parents on salt tolerance should be mined from all chromosomes. We used QTL-sequencing and linkage analysis to detect an overlapping peak interval (**Figure 3A**). Linkage analysis additionally detected *qSDS1* and *qRNC7* on chromosomes 1 and 7, indicating a difference in the allele ratio among the F_2:3_ populations. Interestingly, *OsHsfA7*, located in the *qSDS1* interval, is a heat shock protein. Under salt treatment, rice overexpressing *OsHsfA7* relieved damage symptoms and higher survival rates (Liu et al., 2013). *qSTS4* was a major QTL detected on chromosome 7, contributing to 33.14%, 28.09%, 30.40%, and 29.21% of the phenotypic variations for SDS, RKC, SNC, and SKC, respectively (**Supplementary Table 6**), whereas Na^+^ concentration exerted only a small effect and accounted for 8.12% of the variance for *qRNC7*. It is noteworthy that *qSTS4* and *qRNC7* were not detected in the previous study, probably due to the lack of variations in these two QTLs between the two parents. Therefore, these two loci can be used as new salt-tolerant targets in LD19 for subsequent molecular breeding. Currently, the different functions of alleles of several key genes under salt stress have been confirmed, such as *OsCPK17* and *OsHKT1;5* (Negrão et al., 2013), *HKT1;5* and *HKT2;3* (Mishra et al., 2016), as well as the salt-tolerant alleles obtained from 3000 rice resources(Campbell et al., 2017; Liu et al., 2019). However, these alleles were considered for salt-tolerant breeding of northern japonica rice before the separation of japonica rice in northern China was resolved. In our study, both TX144 and LD19 are northern japonica rice. The results of QTL-seq and linkage analysis suggest that the SNP in the confidence interval causes phenotypic separation, indicating that there are alleles that can distinguish salt tolerance in these two cultivars. In our study, it is unclear whether the SNP difference in the promoter of *OsBBX11* of TX144 and LD19 is the cause of the differential response to salt stress. To elucidate this issue, we plan to clone the alleles of *OsBBX11*, construct expression vectors of plants and yeast, and express these vectors in rice protoplasts, yeast, and *Arabidopsis* to compare the effects of this natural allele variations on gene expression and subcellular localization under salt stress.

The B-box (BBX) gene family, encoding zinc-finger proteins, is an important family of transcription factors in plants. It has several functions, such as response to environmental changes and plant growth and development (Robson et al., 2001; Lira et al., 2020). In rice, the *BBX* genes such as *OsCO3* (Kim et al., 2008), *Hd1* (Zong et al., 2021), *OsCCT19* (Zhang et al., 2015), *DTH2* (Wu et al., 2013), *OsCOL15* (Wu et al., 2018), and *OsBBX14* (Bai et al., 2016) are largely involved in regulating the heather dying stage and rice response to light. However, none of the BBX genes have been characterized to be associated with salinity tolerance in rice. However, several *BBX* genes have been reported to be associated with the regulation of salt tolerance in plants. For instance, the overexpressed *Malus domestica* gene *MdBBX10* (Jackson and Linsley, 2004) enhanced salt tolerance in *Arabidopsis*, as well as *AtBBX5* (Min et al., 2015) and *AtBBX24* (Nagaoka and Takano, 2003) in *Arabidopsis* are induced by salt stress. Although A and B genes,.*OsBBX4* (Liu et al., 2012) and *OsBBX22* (Jain et al., 2007) in rice are induced by salt stress, their salt-tolerance functions have yet to be characterized. Recently, several key genes, such as *OsMIR408*, *OsmiR535*, *OsNAC3*, and *BEARI*, have been intervened by gene knockout technology to alleviate the damage of salt stress in plants (Yue et al., 2020; Zhang et al., 2021; Zhou et al., 2021; Teng et al., 2022). In our study, *OsBBX11* was mapping as a B-box zinc finger family protein (BBX). Although transgenic lines confirmed that OsBBX11 may play a salt-tolerant function by affecting the transport of sodium and potassium ions from underground to aboveground, the underlying molecular mechanism remains elusive. Previous studies have demonstrated that *OsBBX11* was down-regulated by including NAA (a member of the auxin family), GA3 (a gibberellin), and KT (a cytokinin) (Huang et al., 2012). Based on this, the study of the salt tolerance mechanism of *OsBBX11* should consider the effects of ion transport channel proteins or hormone regulation. As a newly identified salt-tolerance regulatory factor, the physiological mechanism of salt-tolerance regulation of *OsBBX11* remains unclear. In addition, the molecular mechanism of promoter components interacting with transcription factors and the protein factors interacting with *OsBBX11* also need further investigation. Moreover, whether hormones are involved in regulating the response to salt stress warrants verification by creating multiple types of transgenic material.

## Conclusion

Based on QTL-seq and high-density linkage map, we identified QTLs associated with seedling salt-tolerance in rice. qRT-PCR analysis showed that the salt-responsive gene *OsBBX11* was a candidate gene in *qSTS4* region. Transgenic plant phenotypic detection confirmed that *OsBBX11* plays a salt-tolerance function by affecting the transport of Na^+^ and K^+^ from underground to aboveground. In addition, its alleles of the salt-specifically induced expression pattern of the Longdao-9 can be an important target for salt-tolerance improvement in rice.

## Data availability statement

The datasets presented in this study can be found in online repositories. The names of the repository/repositories and accession number(s) can be found below: NCBI SRA: SRX18634378, SRR22671406, SRR22671407, and SRR22671408.

## Author contributions

LL, LZC and GHD conceived and designed the research. LMC, JXW, JSZ, TSX and LC participated in data analysis. YL, KL, QJL, TTX, GY and XYW performed material development, sample preparation, and data analysis. LL and SCS wrote the manuscript. SCS and YCL corrected the manuscript. All authors contributed to the article and approved the submitted version.

## Supplementary material

Supplementary Figure 1 A linkage map covering a total genetic distance of 2252.63 cM and 4326 SNP markers in the rice genome with an average distance between Bin markers of 2.03 cM. *qSDS4-1*, *qSKC4*, *qSNC4*, *qRKC4*, *qSDS1* and *qRNC7* were detected based on a threshold of LOD>3.
