# Supplementary figures and images for: OsBBX11 on qSTS4 links to salt tolerance at the seeding stage in Oryza sativa L. ssp. Japonica
Source: Front Plant Sci. 2023 Mar 8;14:1139961. doi: 10.3389/fpls.2023.1139961 (PMC10030886; doi:10.3389/fpls.2023.1139961)

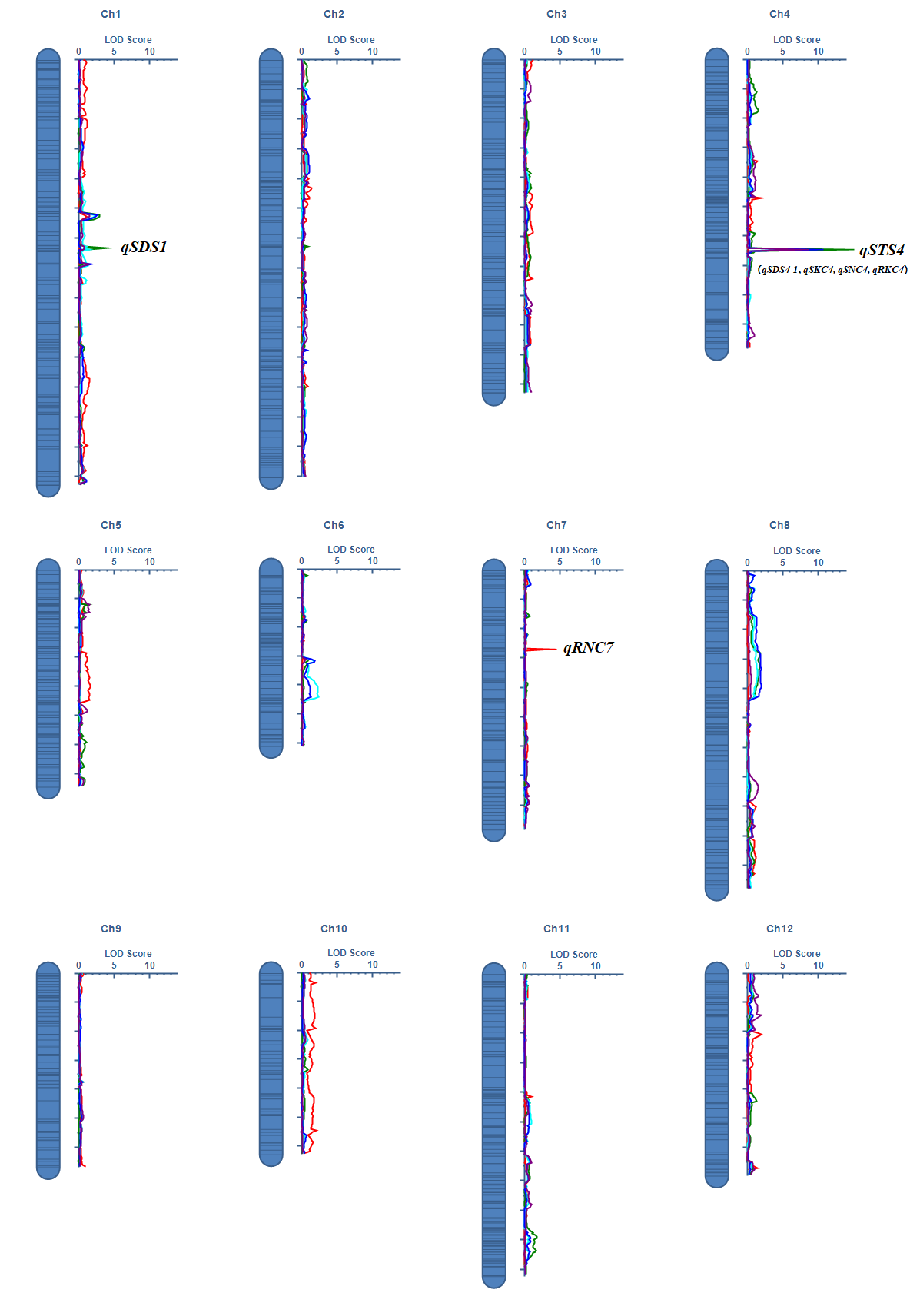

Supplement: Supplementary Figure 1 — A linkage map covering a total genetic distance of 2252.63 cM and 4326 SNP markers in the rice genome with an average distance between Bin markers of 2.03 cM. qSDS4-1, qSKC4, qSNC4, qRKC4, qSDS1 and qRNC7 were detected based on a threshold of LOD>3. [file Image_1.tif]
